# Combined osmotic pretreatment and hot air drying: Evaluation of drying kinetics and quality parameters of adajamir (*Citrus assamensis*)

**DOI:** 10.1016/j.heliyon.2023.e19545

**Published:** 2023-08-26

**Authors:** J. Nudar, M. Roy, S. Ahmed

**Affiliations:** a Department of Food Engineering and Tea Technology, Shahjalal University of Science and Technology, Sylhet, 3114, Bangladesh

**Keywords:** Osmotic treatment, Drying kinetics, Phenolics, Antioxidant, Ascorbic acid, Adajamir

## Abstract

Adajamir (*Citrus assamensis)* is a highly perishable but nutritional fruit. Hot air drying is ubiquitous in food preservation but not quality friendly. However, drying pretreatments play an indispensable role preserving fruits and vegetables. The aim of this study was, therefore, to reveal the hot air drying kinetics of osmotically pretreateated adajamir and investigate the quality parameters (total phenolic contents, antioxidant capacity, and vitamin C). Adajamir slices were subjected to osmotic pretreatment (10% sucrose, 10% fructose, and 2% NaCl), subsequently, dried in a hot air dryer at 50 °C, 30% relative humidity (RH), and with a velocity of 1 ms^−1^. The drying kinetics were studied using three mathematical models: Newtonian model, Henderson and Pabis model, and Page model. The result depicted that effective diffusivity was highest (9.5 ± 0.2^a^ × 10^−6^ m^2^s^−1^) in untreated samples compared to the treated samples, and the Page model was the one with the best fitness to explain the drying behavior. Regarding quality, the pretreatments provided better retention of all quality parameters compared to the untreated samples. In addition, osmotic treatment with sucrose had the best quality retention capability. The study will contribute to the optimization of thermal processing parameters in fruit dehydration. Eventually, this research will expedite future research pertinent to innovative combined drying techniques of citrus fruit.

## 1 Introduction

*Citrus assamensis* is locally known as Adajamir, a rare citrus fruit occurring in Northeast India [1]. It is found in the Khasi Hills region of Meghalaya, the North Cachar area of Assam, and found in the greater Sylhet areas of Bangladesh. It has medicinal properties [2] and is widely used by local tribes of Assam, India. In the Sylhet region of Bangladesh, it is very popular for its typical aromatic flavour like Eucalyptus.

Citrus fruits contain different bioactive compounds and nutrients [3,4]. Due to the existence of vitamin C, phenolic compounds, and natural anti-oxidants in citrus fruits, it has substantial health value [[5], [6], [7], [8]]. However, the major challenge in food processing is preserving the fresh cut after post-harvesting and during processing. Drying is one of the most prominent methods for preserving citrus fruits reducing the moisture content that precludes microbial contamination to make them available all year round [9]. Fruits, vegetables and their products are dried to enhance storage stability, minimize packaging requirements, and reduce transport weight [[10], [11], [12], [13]]. Consequently, common drying techniques are practiced for different foodstuffs, including sun drying, hot air drying, vacuum drying, freeze drying, osmotic drying, and microwave drying. In considering simple equipment, diversified energy utilization, and mass production, hot-air drying is ubiquitous in agro-industrial production [14,15]. However, high temperature and prolonged drying periods contribute to food products' undesirable quality loss and nutritional degradation during the hot air drying [16,17].

Several pre-drying treatments are employed to prevent adverse effects during hot air drying. Such as, osmotic pre-treatment can reduce the moisture content of raw food products before drying. Hypertonic solutions, such as sucrose, fructose, glucose, maltose, corn syrup etc., are used in the osmotic dehydration of food that contributes to the partial removal of moisture from the food body and improves product quality, ensuring the required moisture and solute ratio, reducing thermal stress, and minimizing energy input through the conventional drying period [18]. Numerous studies [[19], [20], [21], [22], [23]] have revealed the effects of osmotic treatments on the moisture transport and physical characteristics of different fruits. But osmotic dehydration may lead to a lower drying rate and moisture diffusion. For instance Ref. [24], reported a decrease of moisture diffusion in sucrose-treated cranberries. But, combined osmotic pretreatment and hot air drying of fruits and vegetables [[25], [26], [27], [28]] have proven to be extremely effective in improving drying rates and persisting the quality.

However, there is a scarcity of knowledge about the combined osmotic-hot air-drying kinetics of adajamir. To the best of our knowledge, no study was conducted to demonstrate the drying kinetics of adajamir. Thus, this work aimed to investigate the effect of osmotic pretreatment on hot air drying characteristics and bioactive compounds of adajamir.

## 2 Materials and methodology

### 2.1 Sample preparation

The fresh ripe Adajamir (*Citrus assamensis*) fruits were collected from Sylhet local wholesale market Bondor Bazar, Sylhet, Bangladesh. Visual imperfect, diseased, and damaged fruits were removed to minimize biological variability. Samples were washed properly with distilled water to remove dirt and stored at 4 °C until the experimental moment. The fruit pulp was sliced into 10 mm thicknesses using a sharp stainless-steel knife and measured with a Vernier Caliper. Different concentrations (ranged from 2 to 10%) of sucrose, fructose, and brine solution were studied, and the concentrations that were closer in terms of moisture removal from fruit pulp were selected for the further experiment of drying kinetics evaluation. Three selected osmotic treatments were applied for 10 min, as explained in Table 1. In each osmotic treatment, the solution to fruit ratio was 10:1 (v/w), according to Ref. [22].

**Table 1:** Different Pretreatment Methods of adajamir.

No	Methods	Pretreatment Process
1	Untreated (UT)	No pretreatment
2	Sucrose Treatment (ST)	Dipped into 10% Sucrose solution for 10 min
3	Fructose Treatment (FT)	Dipped into 10% fructose solution for 10 min
4	Brine Treatment (BT)	Dipped into 2% NaCl solution for 10 min

### 2.2 Hot air drying and drying kinetics

The drying study for either treated or untreated samples was conducted using Constant Temperature and Humidity Chamber (Model: VS-8111H-150). The adajamir samples were dried at 50 °C, 30% Relative Humidity (RH), and an air velocity of 1 ms^−1^. The samples were placed in a thin layer on a stainless-steel tray, and the weight was measured in different intervals until the two consecutive weight difference reached to 0.01g. The dried samples were packaged and stored at −5 °C for further quality analysis.

#### 2.2.1 Modeling of drying kinetics

Several studies were conducted to evaluate the food drying model. Three models were evaluated to determine the most suitable one and determine the drying rate. The models that were used for adajamir drying kinetics are listed in Table 2.

**Table 2:** Available model kinetic expressions used in fruits drying.

Model	Expression	Reference
Newtonian	MR = exp (-K t)	[21]
Henderson and Pabis	MR = a exp (-K t)	[16]
Page	MR = a exp (-K tn)	[29]

*Note.* MR= Moisture Ratio = $\frac{M-M_{e}}{M_{0}-M_{e}}$; M = Moisture content after time, t; M_o_ = Initial moisture content; M_e_ = Equilibrium moisture content; K = Drying Rate Constant; and a, n = Model Constant.

The coefficient of determination (*R*^*2*^) and root mean square error (RMSE) were the statistical parameters considered for selecting the model that best describes the variation in the moisture ratio values of adajamir during the drying process. The model that had the best goodness of fit is the model that has the highest value of *R*^*2*^ and the lowest values of RMSE [30]. According to Ref. [31], (1), (2) represents *R*^*2*^ and RMSE respectively, where $O_{i}$ is the experimental moisture ratio and $J_{i}$ is the predicted moisture ratio at observation *i*, *N* is the number of experimental data points, $\bar{O}$ and $\bar{J}$ are the average sum of the $O_{i}$ and $J_{i}$ respectively.

(1) $$R^{2}={\left[\frac{\sum \left(O_{i}-\bar{O}\right)\left(J_{i}-\bar{J}\right)}{\sqrt{\sum \left(O_{i}-\bar{O}\right)}\sqrt{\sum {\left(J_{i}-\bar{J}\right)}^{2}}}\right]}^{2}$$

(2) $$\text{RMSE}=\left[\frac{\sum {\left(O_{i}-J_{i}\right)}^{2}}{N}\right]$$

#### 2.2.2 Effective diffusivity (D_*eff*_)

Fick's second law is widely used in different research works [15,29,[32], [33], [34]] for the determination of *D*_*eff*_ of fruits. The solution of Fick's second law is summarized in Eqns. (3)–(5) where MR: moisture ratio, *D*_*eff*_: effective moisture diffusivity (m^2^s^−1^) and L: half-thickness (m) of adajamir slices.

(3) $$\text{MR}=\frac{8}{\pi^{2}}\sum_{n=0}^{\infty }\frac{1}{{\left(2n+1\right)}^{2}}\exp\left(-\frac{\pi^{2}\;{\left(2n+1\right)}^{2}}{{4L}^{2}}\right)\;D_{\text{eff}}\;t$$

Eqn. (3) is based on three assumptions: the moisture diffusivity was constant, the adajamir slices represented infinite slab geometry, and the initial moisture distribution was uniform [32,35].

Simplifying Eqn. (3), a straight-line equation was derived as Eqn. (4):

(4) $$\ln\;MR=In\left(\frac{8}{\pi^{2}}\right)-\left(\frac{\pi^{2}}{L^{2}}\;D_{eff}t\right)$$

The plot of experimental drying data in terms of ln (MR) against time (t) gave a straight line with a negative slope (φ) expressed in Eqn. (5):

(5) $$\varphi =\left(\frac{\pi^{2}}{L^{2}}\;D_{eff}\right)$$

### 2.3 Analysis of quality parameters

#### 2.3.1 Determination of total phenolic content (TPC)

According to Ref. [36] 88, TPC was determined by using the Folin-ciocalteu phenol reagent. The absorbance was taken at 765 nm using a UV–Vis Spectrophotometer (Model-T60U, PG Instruments Limited, UK). Gallic acid was used as a standard solution to make the standard curve, and therefore the results of total phenols concentration were expressed in terms of Gallic Acid Equivalent (GAE) in mg/100g of dry matter (DM).

#### 2.3.2 Determination of antioxidant capacity

DPPH (2, 2-diphenyl1-picrylhydrazyl) analysis was performed based on [36] which indicated the radical scavenging activity of adajamir and determined antioxidant capacity. The absorbance was measured at 517 nm using a UV–Vis Spectrophotometer (Model-T60U, PG instruments limited, UK), and Eqn. (6) was used to determine the percentage (%) of the free radical scavenging activity where Aₒ = absorbance of control blank, and A_s_ = absorbance of sample extract:

(6) $$\text{Radical}\;\text{scavenging}\;\text{activity}\left(\%\right)=\left(\frac{Aₒ-Aₛ}{Aₒ}\right)\times 100$$

#### 2.3.3 Determination of vitamin C

The Ascorbic Acid (vitamin C) was determined based on the reduction of 2,6-dichlorophenol-indophenol by ascorbic acid and those based on the reduction of dehydroascorbic acid with 2,4-dinitrophenylhydrazine as described by Ref. [37]. In this method, the dye, is blue in an alkaline solution and red in an acid solution, was reduced by ascorbic acid to a colourless form. The dye factor, mg of ascorbic acid per ml of the dye, was determined by Eqn. (7):

(7) $$Dye\;factor=\frac{Concentration\;of\;ascorbic\;acid\;x\;volume\;of\;ascorbic\;acid}{volume\;of\;titrate}$$

Finally, the amount of vitamin C (mg of ascorbic acid/100 g DM) was estimated using Eqn. (8):

(8) $$\frac{Titre\times Dye\;Factor\times Volume\;made\;up\times 100}{Aliquot\;of\;extract\;taken\;for\;estimation\times Weight\;of\;the\;sample}$$

### 2.4 Statistical analysis

The data were analyzed using Statistical Package for Social Sciences Software (SPSS, version 22.0, IBM). One-way ANOVA was carried out, and post-hoc Turkey's tests were used to determine the significant differences with a confidence interval of 95% (p-value <0.05). All results are reported as mean ± standard deviation of triplicates.

## 3 Results and discussion

### 3.1 Drying kinetics of osmotic pre-treated adajamir

#### 3.1.1 Drying curve

**Fig. 1:**
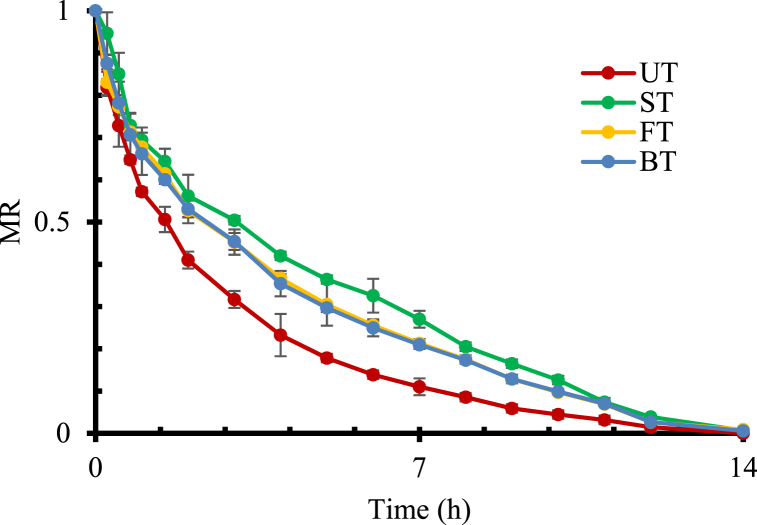
Effect of pre-treatment on the drying curves of adajamir samples.

The initial moisture content of adajamir was estimated 308.6 ± 20.61g/100g DM (Table 3). As indicated by the drying curve (Fig. 1), the Untreated (UT) samples reached in equilibrium moisture condition (EMC) faster than all other treated samples; it took approximately 12 h for UT samples to reach equilibrium moisture content, whereas approximately 14 h for all the treated samples. It was expected that sugar and salt incorporation might lead to surface hardening and a decrease in moisture evaporation rate [10,38,39]. Also, the sugar and salt solution may bind strongly with water molecules that may be responsible for the decrease of driving force for dehydration, the greater will be the rate of evaporation of water into the air and hence increase the drying rate [40]. This result was also comparable with previous research on the drying curve of different fruits and vegetables, such as mango [41], papaya [42], apple, ginger, carrot, and pumpkin [10], Chilean papaya [43].

**Table 3:** Moisture content and quality parameters of fresh adajamir.

Characteristics	Average Standard Deviation
Moisture content (g/100g DM)	308 ± 20
Total Phenolic Content (mg GAE/100g DM)	11.2 ± 0.5
DPPH Free Radical Scavenging Activity (%)	73 ± 3
Vitamin C (mg ascorbic acid/100g DM)	269 ± 5

#### 3.1.2 Effective diffusivity

*D*_*eff*_ was higher in UT samples than in other osmotic-treated samples (Table 4). As expected, the mass transfer of osmotic treated fruits and vegetable generally decreases due to the incorporation of osmotic solutes into the samples, significantly decreasing their porosity [43]. An observation was found in previous researches where the authors found the values of *D*_*eff*_ for untreated samples were higher than that of osmotic pre-treated samples, such [21,44] obtained the value of *D*_*eff*_ for treated papayas 1.03-1.78 × 10^−9^ m^2^ s^−1^ and 3.57 × 10^−6^ m^2^ s^−1^ respectively [45]; presented *D*_*eff*_ for osmotic pre-treated pears 1.87-8.12 × 10^−10^ m^2^ s^−1^.

**Table 4:** Regression coefficient and RMSE values of different drying models and effective diffusivity of Adajamir at 50 °C.

Temperature °C	Treatment		*R*^*2*^			RMSE		Effective Diffusivity ( × 10^−6^ m^2^s^−1^)
		Newton	Herderson and Pabis	Page	Newton	Herderson and Pabis	Page	
	ST	0.9264	−0.0173	0.9775	0.1289	0.4794	0.0711	9.1 ± 0.6^ab^
	FT	0.9664	0.93599	0.9959	0.0806	0.1114	0.0279	8.8 ± 0.6^ab^
50 °C	BT	0.9648	0.83425	0.9806	0.0842	0.1829	0.0624	8.2 ± 0.2^b^
	UT	0.9515	0.41795	0.9781	0.0999	0.3461	0.0670	9.5 ± 0.2^a^

#### 3.1.3 Fitting of the drying model

The fitting of the drying model based on the calculation of $R^{2}$ and RMSE summarized in Table 4. The result indicated that the values ranged from −0.0173 to 0.9959 for $R^{2}$ and 0.0279 to 0.4794 for RMSE. According to the criteria of the highest $R^{2}$ and the lowest RMSE the Page model was the best-fitted model for both treated and untreated samples. Based on the literature, the Page model is one of the most suitable for describing the drying behavior of various fruits and vegetables [34,46,47].

### 3.2 Quality parameters

#### 3.2.1 Total phenolic content (TPC)

**Fig. 2:**
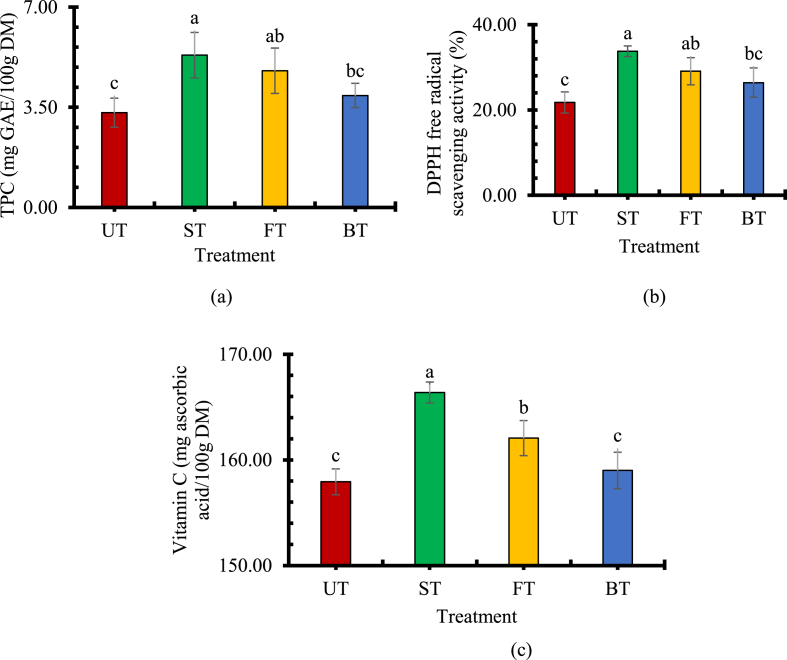
**a)** TPC at EMC for osmotic-treated and untreated adajamir; **b)** DPPH free radical scavenging activity at EMC for osmotic-treated and untreated adajamir; **c)** Vitamin C at EMC for osmotic-treated and untreated adajamir. Different letters on the bars show a significant difference (p < 0.05).

The total phenolic content in fresh adajamir was estimated at 11.2 ± 0.5 mg GAE/100g DM (Table 3). The result depicted that the loss of TPC is significantly higher (approximately 70%) for UT samples compared to ST (approximately 52%) and FT (approximately 57%) samples after reaching EMC (Fig. 2a). In addition, the loss is higher in UT samples than that of BT (approximately 65%) samples. Thus, ST retained the highest TPC of all other samples. The mass transfer rate was higher in UT samples compared to the treated samples due to the barrier effects of osmotic treatment that resulted in the highest TPC loss in UT. However [18,48], and [49] found the lowest TPC in Mulberry, nutmeg pericarp, and blueberries, respectively, treated with sucrose solution for 3hr that opposed the present study where the treatment was conducted for only 10min. The higher the osmotic treatment time, the higher the loss in TPC occurs due to the migration of phenolic contents into the osmotic solution by the osmotic driving force [50].Therefore, treatment with sucrose for 10min provided good TPC protection. Moreover, the variation in TPC loss among the osmotic treatment due to the different diffusion rates of the ST, FT, and BT solutions might lead the TPC to come out from the sample differently.

#### 3.2.2 Antioxidant capacity

The effect of pretreatment on antioxidant capacity was determined with complement to the DPPH free radical scavenging activity by the adajamir samples (Fig. 2b). Approximately 73 ± 3% DPPH free radical scavenging activity was recorded in fresh adajamir sample (Table 3). Like TPC, approximately 70% of DPPH free radical scavenging activity decreased in UT samples which were significantly lower compared to ST (approximately 54%) and FT (approximately 60%) samples at EMC. A similar observation was also delineated by Ref. [51] for osmotic pretreated papaya. The present study further revealed that the decrease of DPPH free radical scavenging activity was the lowest in ST samples. An osmotic solution like sucrose has a great barrier property to the outflow of the antioxidant compounds [11,52,53]. Therefore, osmotic treatment by sucrose had the greatest retention of antioxidant capacity.

**Fig. 3:**
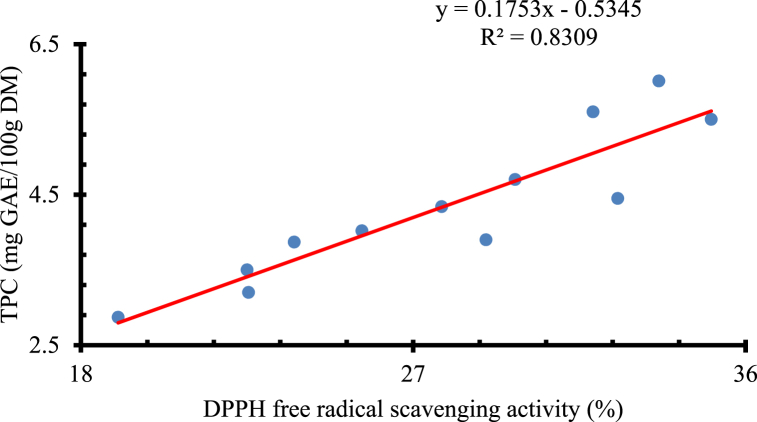
Correlation (*r*) between TPC and DPPH free radical scavenging activity for osmotic-treated and untreated adajamir at p<0.05.

However, a strong positive correlation was found between TPC and DPPH free radical scavenging activity with a Pearson-Correlation coefficient of *R*^*2*^ = 0.8309 (Fig. 3). This relationship was comparable with [49] who found a strong correlation (*R*^*2*^ = 0.97) between TPC and DPPH free radical scavenging activity of osmotically dehydrated nutmeg pericarp [54,55]. reported a strong correlation between TPC and antioxidant capacity in different eggplants and lychee pericarp, respectively. Bioactive compounds like phenolic contents have antioxidant properties [56], that might attribute to this correlation.

#### 3.2.3 Vitamin C

Vitamin C content in adajamir is represented as ascorbic acid equivalent (Fig. 2c). The fresh adajamir contained 269.84 ± 5.44 mg ascorbic acid/100g DM (Table 3) [57]. reported that Vitamin C is highly heat sensitive and water-soluble. Moreover, hot air drying causes oxidation, thermal destruction, and leaching of ascorbic acid in fruits and vegetables [45,58,59]. On the other hand, the osmotic treatment allows a portion of moisture to be leached out from the sample at room temperature, protects vitamin C. Also, the solute is migrated to the osmotic solution [21], which might have a barrier effect to leach out vitamin C from the sample during hot air drying. Thus, the UT adajamir sample was expected to lose higher vitamin C than the osmotic-treated sample. The result depicted that the loss of vitamin C was significantly lowest (approximately 38%) in ST samples at EMC while the loss was highest (approximately 42%) in UT samples [60]. also found a similar result for papaya, and [61] for pineapple, orange, watermelon, and tomato.

## 4 Conclusion

Osmotic pre-treatment was employed to observe the drying kinetics and quality parameters of adajamir. The Page model was the best-fitted model to describe the drying kinetics for treated and untreated adajamir. Osmotic treatment caused a barrier that led to lower moisture diffusivity and took 14hr to reach equilibrium moisture condition whereas the untreated sample took 12hr. On the other hand, the osmotic barrier effect ensures better quality retention. Therefore, it can be revealed that the osmotic pretreatment of adajamir needs a longer drying period though it can retain more quality compared with the untreated one. However, a positive correlation was found between the TPC and DPPH free radical scavenging activity in adajamir samples. It can be concluded that the osmotic pretreatment by sucrose is the best treatment before the hot air drying at 50 °C and 30% RH to retain the highest TPC, DPPH free radical scavenging activity, and vitamin C in adajamir. The findings of the present study will open a new window to preserve adajamir using thermal treatment by retaining most quality parameters unchanged. Also, the study will inspire the food and beverage industries to design new food products using adajamir pulp with extended shelf life.

## Author contribution statement

Jannatul Nudar: Performed the experiment; wrote the paper

Mukta Roy: Conceived and designed the experiment; Contributed reagents, materials, analysis tools or data

Shafaet Ahmed: Conceived and designed the experiment; Analyzed and interpreted the data; wrote the paper

## Data availability statement

Data will be made available on request.

## Declaration of competing interest

The authors declare that they have no known competing financial interests or personal relationships that could have appeared to influence the work reported in this paper.
